# Correction: The effect of balint practice on reducing stress, anxiety and depression levels of psychiatric nurses and improving empathy level

**DOI:** 10.1186/s12912-024-02425-7

**Published:** 2024-10-11

**Authors:** Yunnan Mao, Fenghong Zhang, Yanyan Wang, Qiuyue Hu, Lingyun Fan

**Affiliations:** The Second People’s Hospital of Gansu Province, 730030 Lanzhou, P. R. China

**Correction: BMC Nursing (2024) 23:554**

10.1186/s12912-024-02189-0

The original article contains the following errors which the authors would like to clarify:

1. Directly under the heading, The Jefferson Scale of Empathy-health professionals, JSE-HP, the first sentence should be modified to include the following additional text:

Using the JSE-HP Chinese version score (the JSE-HP scale was developed by Dr. Mohammadreza Hojat and his research team at Jefferson University’s Center for Medical Education and Healthcare Research [1]), the scale includes a total of 20 items […].

2. Later on in the same paragraph, the number ‘17’ should instead be ’20’.
